# Clinical study of Gene-Eden-VIR/Novirin in genital herpes: suppressive treatment safely decreases the duration of outbreaks in both severe and mild cases

**DOI:** 10.1186/s40169-016-0121-6

**Published:** 2016-10-20

**Authors:** Hanan Polansky, Edan Itzkovitz, Adrian Javaherian

**Affiliations:** The Center for the Biology of Chronic Disease (CBCD), 616 Corporate Way, Suite 2-3665, Valley Cottage, NY 10989 USA

**Keywords:** Acyclovir, Valacyclovir, Famciclovir, Natural treatment, Genital herpes, Outbreaks

## Abstract

**Background:**

We conducted a clinical study that tested the effect of suppressive treatment with the botanical product Gene-Eden-VIR/Novirin on genital herpes. Our previous paper showed that the treatment decreased the number of genital herpes outbreaks without any side effects. It also showed that the clinical effects of Gene-Eden-VIR/Novirin are mostly better than those reported in the studies that tested acyclovir, valacyclovir, and famciclovir. The current paper reports the effect of suppressive treatment with Gene-Eden-VIR/Novirin on the duration of outbreaks, in severe and mild genital herpes cases.

**Methods:**

The framework was a retrospective chart review. The population included 137 participants. The treatment was 1–4 capsules per day. The duration of treatment was 2–48 months. The study included three controls: baseline, no-treatment, and dose–response.

**Results:**

The treatment decreased the duration of outbreaks in 87 % of participants and decreased the mean duration of outbreaks from 8.77 days and 6.7 days in the control groups to 2.87 days in the treatment group (P < 0.001, both groups). All participants reported no adverse experiences.

**Conclusions:**

This paper shows that suppressive treatment with Gene-Eden-VIR/Novirin decreased the duration of genital herpes outbreaks, in both severe and mild cases, without any side effects. Based on the results reported in this and our previous paper, we recommend suppressive treatment with Gene-Eden-VIR/Novirin as a natural alternative to both suppressive and episodic treatments with current drugs, in both severe and mild genital herpes cases.

*Trial registration* ClinicalTrials.gov NCT02715752 Registered 17 March 2016 Retrospectively Registered

## Background

Genital herpes is one of the most important public health problems [1]. It is a sexually transmitted infection caused by two viruses: the Herpes Simplex Virus Type 2 (HSV-2), and Herpes Simplex Virus Type 1 (HSV-1) [2]. In the United States, one in four persons 30 years of age or older has HSV-2 [3].

Three drugs are used in the treatment of genital herpes: acyclovir (ACV), valacyclovir (VACV, a prodrug of ACV), and famciclovir (FCV, a prodrug of penciclovir) [4, 5]. ACV was approved by the Food and Drug Administration (FDA) in 1984, VACV in 1995, and FCV in 1994. These drugs use modified nucleosides, or their prodrugs [6]. The drugs inhibit the activity of the viral DNA polymerase, which is the main replication enzyme of the virus.

All three drugs are used in both episodic and suppressive therapy. Episodic therapy uses short term, self-administration of the drug during outbreaks. The main objective of episodic therapy is to decrease the duration of the outbreaks [7]. Suppressive therapy involves long term, daily administration of the drug before the onset of outbreaks. The main objective of suppressive therapy is to decrease the number of outbreaks [8].

All three drugs are associated with known adverse effects. The most frequent ones, according to the product inserts, are nausea, diarrhea, and headache, for ACV, headache, nausea, and abdominal pain, for VACV, and headache, nausea and dizziness, for FCV.

The medical literature also reports non-common adverse events. For instance, Yavuz et al. reported that a 78-year-old female with normal baseline renal function, and no contributing possible nephrotoxic factors, developed irreversible renal dysfunction after oral ACV treatment [9]. Becker et al. reported that a patient developed rapidly progressive acute renal failure with concomitant mental status changes following treatment with high-dose parenteral ACV [10]. Another uncommon, but serious side effect of ACV treatment, is neurotoxicity that may lead to confusion, hallucinations, seizures and obtundation [11].

Le Cleach et al. conducted a meta-analysis of 26 clinical studies that tested the effectiveness and safety of the three oral antiviral drugs [2]. Interestingly, they found that only 8 out of the 26 studies, or less than a third, reported the number of withdrawals due to harms. These 8 studies reported 14 withdrawals due to harms in the placebo or no treatment groups, and 31 withdrawals in the antiviral groups. This is a 121 % increase in the number of withdrawals due to harms. In addition, only four, or 16 % of the 26 studies, reported safety data in the form of the total number of adverse events. These four studies reported 115 adverse events in 291 participants in the placebo or no treatment groups (40 %), including three serious adverse events, two renal signs, and one fatal pneumonia. In the antiviral groups, they reported 331 adverse events in 561 participants (59 %), including three serious adverse events, one hypertension crisis, one intestinal obstruction, and one angor. In other words, the participants treated with the antiviral drugs reported 48 % more adverse events relative to the non-treated or placebo-treated participants.

Lam et al. analyzed a cohort of 76,269 patients who received acyclovir or valacyclovir, and 84,646 who received famciclovir [12]. The results showed that 0.27 % of those treated with ACV or VACV, and 0.28 % of those treated with FCV, were hospitalized with acute kidney injury (AKI).

It is well accepted that adverse effects are dose-dependent. Therefore, to minimize the risk of adverse effects whenever possible, the medical community decided to recommend using these drugs in short term, or episodic therapy, in mild cases, and long term, or suppressive therapy, in severe cases. Clinical studies have shown that episodic treatment with these drugs can shorten the time to lesion healing by 1–2 days [13–15]. Clinical studies also showed that suppressive treatment for a period of 4–12 months can decrease the number of outbreaks, such that about half of patients remain recurrence-free, and the other half show a 70–80 % decrease in the frequency of their outbreaks [16, 17].

It is interesting that this practice is so ingrained that it had a profound effect on the clinical studies that tested these drugs. Surprisingly, most of the clinical studies that tested suppressive treatments measured the effect on the number of outbreaks, but not their effect on the duration of outbreaks [7, 8, 17, 18].

Gene-Eden-VIR/Novirin is a patented botanical product that consists of five natural ingredients: quercetin 100 mg, green tea extract 150 mg, cinnamon extract 50 mg, selenium 100 mcg, and licorice extract 25 mg. Gene-Eden-VIR/Novirin was developed to target latent viruses including HSV, HPV, CMV and EBV, and diminish their deleterious effect on the host, as explained by the Microcompetition theory [19–21].

The scientists who developed the Gene-Eden-VIR/Novirin formula used a unique scientific tool, Computer Intuition, a proprietary psycholinguistic-based, data-mining program that analyzes scientific text [22]. The objective was to identify natural ingredients found in the scientific literature that have a strong antiviral effect against the most common viruses. To achieve the objective, the scientists used the computer program to analyze more than 50,000 papers. The results assisted the developers in selecting the best ingredients and the most effective dosages.

Gene-Eden-VIR/Novirin was introduced in the marketplace at the end of 2009. A post-marketing clinical study conducted at the Center for the Biology of Chronic Disease (CBCD) showed that Gene-Eden-VIR/Novirin is antiviral [22]. Another post-marketing clinical study showed that Gene-Eden-VIR/Novirin safely decreased the feeling of fatigue in individuals infected with a latent virus [23].

Our previous paper showed that suppressive treatment with Gene-Eden-VIR/Novirin decreased the number of genital herpes outbreaks without any side effects [24]. It also showed that the clinical effects of Gene-Eden-VIR/Novirin are mostly better than those reported in the studies that tested acyclovir, valacyclovir, and famciclovir. The current paper tested the effect of suppressive treatment with Gene-Eden-VIR/Novirin on the duration of outbreaks, in severe and mild genital herpes cases.

## Methods

### Objective

The objective of this clinical study was to test the effect of suppressive treatment with Gene-Eden-VIR/Novirin on the duration of genital herpes outbreaks, and possibly offer a suppressive treatment as an alternative to episodic treatment with current drugs in mild genital herpes cases.

### Framework

The framework was a retrospective chart review. The company that sells Gene-Eden-VIR/Novirin, Lilac Corp., provides a service to the customers who purchase the products. The service consists of tracking the changes in the customers’ health while using the products. The company is using a questionnaire called the Natural Origin Treatment Clinical Questionnaire (NotCiq). The data is collected over the phone by professional interviewers in a single session. The NotCiq questionnaire is a patient reported outcome (PRO) instrument. These sessions produced the charts that were analyzed in this study. More details on the NotCiq questionnaire is available in our previous study [22].

### Randomization

A random selection of charts, collected during a 2-month period, October and November 2015, were analyzed. The 2 months were randomly selected.

### Treatment

The treatment was 1, 2, 3, or 4 capsules of Gene-Eden-VIR/Novirin per day. The duration of treatment ranged from 2 to 48 months. The mean duration of treatment was 12 months.

A total of 137 participants were included in the study. They composed two groups. The treatment group consisting of 117 participants and the no-treatment control group consisting of 20 participants. The study included three FDA recommended controls: baseline control, consisting of 117 participants, a no-treatment control, consisting of 20 participants, and a dose–response concurrent control, consisting of 99 participants. The baseline control included participants “before” treatment, or pre-treatment. The no-treatment control included participants who have purchased Gene-Eden-VIR/Novirin in the last 6 weeks. The dose response concurrent control included 99 participants out of the treatment group.

### Outcome measures

The NotCiq questionnaire for genital herpes has several sections: a section on gender, age, and ethnicity of the participants; a section on the duration of treatment, dosage, and adverse experiences; a section on diagnosis and type of symptoms; a section on the duration of outbreaks; and a section on symptom severity, interference with daily life, and pain. The questionnaire used both open- and closed-ended questions. The answers to the closed-ended questions were on a scale of 1–7.

The answers to the NotCiq questionnaire were collected over the phone by four independent interviewers who specialize in outbound call services. The interviewers were blinded to the objective of the study.

### Efficacy

The primary efficacy end point was the duration of outbreaks, defined as the number of days between initiation and complete resolution of all symptoms and signs. The secondary efficacy end points were the severity of symptoms, interference with daily life, and level of pain.

### Safety

The participants’ reports of adverse events were collected and analyzed.

### Population

Participants who were using Gene-Eden-VIR/Novirin for other purposes, such as treatment for cancer, chronic diseases, and hypertension, were excluded.

Participants concurrently taking antiviral medications, including ACV, VACV, or FCV, as suppressive or episodic treatment, were also excluded. The final list of participants consisted of 137 men and women aged ≥18 years with at least one genital herpes outbreak per year.

### Statistical analysis

The statistical difference between the responses of pre-treatment and post-treatment was calculated. The statistical tests were performed in a then-test model for users. Statistical analysis was performed using a one-tail *t* test assuming unequal variances. P ≤ 0.05 was considered as statistically significant.

## Results

### Population characteristics

A total of 137 participants were included in the study. Table 1 summarizes the demographic and clinical characteristics of these participants (Table 1).

**Table 1:** Demographics and clinical characteristics

	Treatment group	No-treatment group
Age average (years)	50	44
20–40	28 (23.9 %)	8 (40 %)
41–50	30 (25.6 %)	4 (20 %)
51–60	34 (29.1 %)	6 (30 %)
61–80	25 (21.4 %)	2 (10 %)
Gender—number (%)		
Male	61 (52.1 %)	8 (40 %)
Female	56 (47.9 %)	12 (60 %)
Race—number (%)		
African American	31 (26.5 %)	9 (45 %)
Caucasian	66 (56.4 %)	7 (35 %)
Hispanic	11 (9.4 %)	1 (5 %)
Other	9 (7.7 %)	3 (15 %)
Years since diagnosis by physician	0.5–40 (range), 10.7 (mean), 5.0 (median)	0.5–17 (range), 7.3 (mean), 4.5 (median)
Years since initial episode	0.5–48 (range), 13.6 (mean), 9.0 (median)	0.5–17 (range), 11.4 (mean), 10.3 (median)
Diagnosed by physician—number (%)	91 (77.8 %)	17 (85 %)
Lab test performed (out of those diagnosed by a physician)—number (%)	60 (65.9 %)	9 (52.9 %)
Symptoms of infection—number (%)		
Genital blisters/ulcers	102 (87.2 %)	18 (90 %)
Anal blisters/ulcers	21 (17.9 %)	5 (25 %)
Burning feeling while urinating	28 (23.9 %)	4 (20 %)
Local pain	83 (70.9 %)	16 (80 %)
Genital discharge	14 (12.0 %)	1 (5 %)
General discomfort	64 (54.7 %)	11 (55 %)
Light sensitivity	22 (18.8 %)	2 (10 %)
Genital tingling sensation	80 (68.4 %)	13 (65 %)
Genital itching sensation	80 (68.4 %)	13 (65 %)
Flu-like symptoms	51 (43.6 %)	8 (40 %)
Duration of treatment (months)—number (%)		
2–3	22 (16.2 %)	N/A
4–6	24 (17.6 %)	N/A
8–12	31 (22.8 %)	N/A
16–24	25 (18.4 %)	N/A
25–48	14 (10.3 %)	N/A
Dosage of treatment (capsules per day)—number (%)		
1	40 (31.2 %)	N/A
2	72 (56.3 %)	N/A
3	5 (3.9 %)	N/A
4	11 (8.6 %)	N/A

### Primary efficacy end point

**Fig. 1:**
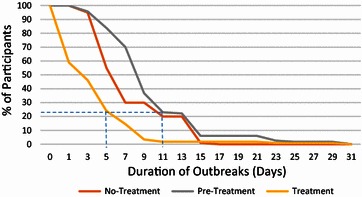
*Graph* showing the percentage of participants versus duration of outbreaks in the treatment, pre-treatment and no-treatment groups

Out of the 117 participants in the treatment group who used Gene-Eden-VIR/Novirin for at least 2 months (see section on duration of treatment effect for explanation), 102 (87 %) reported a decrease in the duration of outbreaks. The mean duration of outbreaks decreased from 8.77 to 6.70 days, in the pre-treatment and no-treatment control groups, respectively, to 2.87 days in the treatment group (P < 0.001 for both groups) (Table 2). This is a decrease of 5.9 and 3.83 days, respectively. The median duration of outbreaks decreased from 7.00 to 5.00 days, in the pre-treatment and no-treatment control groups, respectively, to 2.00 days in the treatment group (Table 2). In 75 % of the participants, the duration of outbreaks decreased to less than 5 days compared to 10.5 and 11 days in the no-treatment and pre-treatment groups, respectively (Fig. 1). Out of the 117 participants in the treatment group, 48 (41 %) reported no outbreaks.

**Table 2:** Summary of efficacy endpoints in current study

N	No-Tx Ctrl	Pre-Tx Ctrl	Tx	P value
	20	117	117	–
Mean duration of outbreaks (days)	6.70	8.77	2.87	P < 0.001 (No-Tx Ctrl)
				P < 0.0001 (Pre-Tx Ctrl)
Median duration of outbreaks (days)	5.00	7.00	2.00	–
On a scale of 1–7^a^	3.95	3.35	5.82	P < 0.0001 (both controls)

*Tx* treatment

^a^Where 1 is “very long time” and 7 is “didn’t have symptoms”

To test the internal consistency of the participants’ responses, the following two questions were included: “How long did your symptoms last, before taking Gene-Eden-VIR/Novirin?” and “How long did your symptoms last, after taking Gene-Eden-VIR/Novirin?”, using a scale from 1–7, where 1 is “very long time” and 7 is “didn’t have symptoms”. The mean scores were 3.35 and 5.82 for the “before” and “after” taking Gene-Eden-VIR/Novirin questions, respectively (P < 0.0001) (Table 2). The results showed that both measures, the one that asks the participant to count the number of days he or she experienced symptoms, and the one that asks to represent the duration of outbreaks using a scale, generated consistent results.

To test for possible differences between patients with a different severity level we compared the change from pre-treatment to post-treatment in participants who reported less than 6 episodes and those who reported at least 6 episodes per year. We compared the change (Δ) in the duration of outbreaks from pre-treatment to post-treatment. No difference was detected between the two groups (P = 0.32; data not shown). To check for consistency, we also tested this difference using the participants’ answers using the 1–7 scale. No difference was found in these answers either (P = 0.28; data not shown).

We also tested the effect of different dosages. We compared the participants who took 1 capsule (N = 36) to those who took 2 capsules (N = 63) per day. The mean duration of outbreaks (in days) before treatment was 8.30 and 8.20 days, respectively (P = 0.45). We than analyzed the duration of outbreaks following treatment in both groups. The mean duration of outbreaks decreased to 2.94 and 1.98 days in the group that took 1 capsule and 2 capsules, respectively (P = 0.058). These results have borderline significance for the existence of a dose effect. We could not test for a dose effect participants that took more than 2 capsules per day since the number of participants who took 3 or 4 capsules per day was too small for statistical analysis.

Finally, we also tested the effect of diagnosis. The data included two types of diagnosis, diagnosis by a physician, and self-diagnosis. The mean duration of outbreaks were 8.65 and 2.82 days during the pre-treatment and treatment period, respectively (P < 0.0001), in participants diagnosed by a physician, and 9.19 and 3.04 days, respectively (P < 0.0001), in self-diagnosed participants. The decrease in duration of outbreaks in these two groups are 5.83 and 6.15 days, respectively. There was no statistically significant difference between the two groups in the pre-treatment and treatment periods (P = 0.32 and P = 0.39, respectively). These results show that the method of diagnosis has no effect.

### Secondary efficacy end points

Severity of symptoms was measured on a scale from 1 to 7, where 1 is “very bad (severe)” and 7 is “not bad at all.” Out of the 115 participants who reported some severity of their symptoms, 101 (88 %) reported a decrease in severity, and 54 (47 %) reported a complete recovery on the severity scale, that is a change from a score of 1–6 to a score of 7 on this scale. A statistical analysis showed that the mean score increased from 3.5 to 3.05 for the no-treatment and pre-treatment control groups, respectively, to 5.98 for the treatment group (P < 0.0001 for both groups) (Table 3).

**Table 3:** Secondary efficacy endpoints in the current study

	No-Tx Ctrl*	Pre-Tx Ctrl*	Tx	P value
Severity	3.50 (N = 18)	3.05 (N = 115)	5.98 (N = 115)	P < 0.0001 (both controls)
On a scale of 1–7^a^				
Interference with daily life	3.71 (N = 14)	3.20 (N = 59)	6.14 (N = 59)	P < 0.0001 (both controls)
On a scale of 1–7^b^				
Pain	3.74 (N = 19)	3.13 (N = 105)	5.84 (N = 105)	P < 0.0001 (both controls)
On a scale of 1–7^c^				

*Tx* treatment

* We excluded those participants that had 7 in in the pre-treatment or no-treatment control groups, or did not respond to the question, that is, we excluded those that did not have the specific symptom

^a^Where 1 is “very bad” and 7 is “not bad at all”

^b^Where 1 is “interfered all the time” and 7 is “did not interfere”

^c^Where 1 is “very painful” and 7 is “no pain at all”

Interference of symptoms with daily life was also measured on a scale from 1 to 7, where 1 is “interfered all the time” and 7 is “did not interfere at all.” Out of the 59 participants who reported some interference with their daily life, 52 (88 %) reported a decrease in this interference, and 31 (53 %) reported a complete recovery on the inference scale. A statistical analysis showed that the mean score increased from 3.71 to 3.20 for the no-treatment and pre-treatment control groups, respectively, to 6.14 (P < 0.0001 for both groups) (Table 3).

Finally, the level of pain experienced during an outbreak was also measured on a scale from 1 to 7, where 1 is “very painful” and 7 is “not painful at all.” Out of the 105 who reported having painful lesions, 92 (87 %) reported a decrease in the level of pain, and 47 (45 %) reported complete recovery from pain. A statistical analysis showed that the mean score increased from 3.74 to 3.13 for the no-treatment and pre-treatment control groups, respectively, to 5.84 (P < 0.0001 for both groups) (Table 3).

### Adverse effects

All participants reported no adverse effects.

## Discussion

This study showed that suppressive treatment with Gene-Eden-VIR/Novirin decreased the duration of genital herpes outbreaks. In addition, the treatment decreased the severity of genital herpes symptoms, the interference of symptoms with daily life, and the level of pain experienced during an outbreak. The results also showed a dose effect. Treatment with 2 capsules per day decreased the duration of outbreaks more than 1 capsule per day. Finally, the results showed that suppressive treatment with Gene-Eden-VIR/Novirin had no side effects, that is, suppressive treatment with Gene-Eden-VIR/Novirin is safe.

Our study has some methodological advantages. The study tested the effect of the treatment over a wide range of durations, from 2 to 48 months, with an average of 12 months. Moreover, 23 % of the participants in our study took Gene-Eden-VIR/Novirin for at least 2 years, and some, for as long as 4 years. The use of a range of durations, rather than a single duration, made it possible to test for a duration of treatment effect.

Another methodological advantage is using two types of questions to gather information about the duration of outbreaks. The first type asked the participants to count the number of days of a typical outbreak, and the second asked them to rate the duration of their outbreaks on a 1–7 scale. Using these two types, we verified the consistency in the participants’ answers.

Yet another methodological advantage is the measurement of the burden of disease. Our study tested a suppressive, or long term treatment, yet, unlike most clinical studies of ACV, VACV, and FCV, it measured the effect on the duration of outbreaks, not the number of outbreaks. If we define the burden of genital herpes as the total number of days with outbreaks, then the burden of genital herpes equals the average duration of an outbreak times the number of outbreaks. Since we could not find clinical studies of suppressive treatments that measured the effect on duration of outbreaks, and clinical studies of episodic treatments that measured the effect on the number of outbreaks, it seems that no previous studies measured the effect of ACV, VACV, and FCV on the burden of the disease. Our paper reports the effect of Gene-Eden-VIR/Novirin on the duration of outbreaks. Polansky et al. [24] reports the effect of Gene-Eden-VIR/Novirin on the number of outbreaks. By multiplying the results in both papers, one can calculate, for the first time, the effect of a treatment on the burden of genital herpes.

This clinical study did not include a placebo control. The FDA guidance lists six types of controls: (1) Placebo Concurrent Control, (2) No-treatment Concurrent Control, (3) Dose–response Concurrent Control, (4) Active (Positive) Concurrent Control, (5) External Control (including Historical Control and baseline-controlled studies), and (6) Multiple Control Groups [25]. This clinical study included three controls recommended by the FDA: a no-treatment concurrent control, a dose–response concurrent control, and a baseline control, a type of external control.

This study used patient-reported outcomes (PROs). PROs are extensively used for collecting clinical data [26]. In fact, out of the 96,736 clinical trials registered in the ClinicalTrials.gov data base between November 2007 and December 2013, 26,337, or 27 %, used at least one PRO measure [27]. In addition, past studies showed that PROs had a significant role in the development and evaluation of new medicines [28]. According to the FDA, PROs are a valid and valuable source for measuring the efficacy of new drugs. They are reliable enough to warrant an approval of a label claim for a new drug. From the years 1997 to 2002, the FDA approved 23 new drugs based on results obtained in studies that used only PRO endpoints. They include six anti-migraine products (Amerge^®^, Ax-ert^®^), several anti-epileptics (Gabitril^®^, Keppra^®^), and a variety of other therapy classes (Tamiflu^®^, Relenza^®^). The FDA regards PROs as a valid and valuable source of data. The scientific community also believes that PROs are valid and useful. Many major journals published clinical studies that use patient reported outcomes. The trust of the FDA and the scientific community in PROs should convince the medical community, and specifically, doctors, to trust studies that use PROs when evaluating the benefits of new treatments.

Due to nature of PRO’s, a possible limitation in this type of study might be the subjective reports by the participants. One might argue that the participants’ have evaluated the effect of Gene-Eden-VIR/Novirin on symptoms, which are unrelated to their infection. To address this question, we compared the symptoms as reported by the participants to the standard signs and symptoms as reported in the literature [29]. The comparison clearly showed that the reported symptoms and the major standard symptoms of genital herpes as found in the literature overlapped (data not shown).

The framework of this clinical study is nontraditional. It tested a suppressive, or long term treatment, in both severe and mild genital herpes cases. Such a framework deviates from the common medical practice. The reason for deviating was our dissatisfaction with the health benefits of the current medical practice. Gene-Eden-VIR/Novirin has an excellent safety profile. In a previous study, we reported that long term daily use of Gene-Eden-VIR/Novirin has no side effects [22]. This inspired us to test an alternative practice, a suppressive, rather than episodic treatment, with Gene-Eden-VIR/Novirin, in both severe and mild cases of genital herpes. We were happy to discover that our bet paid off. We believe that the results in our study prove the value of this treatment. It is interesting that the Gene-Eden-VIR/Novirin alternative is consistent with the preference of patients. Romanowski et al. reported that the subjects in their study, who were under suppressive treatment, felt that it offered a better control of the disease, and a more convenient option [30].

## Conclusions

This paper shows that suppressive treatment with Gene-Eden-VIR/Novirin decreased the duration of genital herpes outbreaks, in severe and mild cases, without side effects. Based on the results reported in this and our previous paper, we recommend suppressive treatment with Gene-Eden-VIR/Novirin as a natural alternative to both suppressive and episodic treatments with current drugs, in both severe and mild genital herpes cases.

## Abbreviations

HSV-1: Herpes Simplex Virus Type 1
HSV-2: Herpes Simplex Virus Type 2
ACV: acyclovir
VACV: valacyclovir
FCV: famciclovir
AKI: acute kidney injury
CBCD: The Center for the Biology of Chronic Disease
PRO: patient reported outcome
NotCiq: Natural Origin Treatment Clinical Questionnaire
EGCG: epigallocatechin gallate
AMPK: AMP-dependent kinase
MPO: myeloperoxidase
MDA: malondialdehyde
RS: reactive species
SOD: superoxide dismutase

## References

[1] Chosidow O, Drouault Y, Leconte-Veyriac F (2001). Famciclovir vs. aciclovir in immunocompetent patients with recurrent genital herpes infections: a parallel-group, randomized, double-blind clinical trial. Br J Dermatol.

[2] LeCleach L, Trinquart L, Do G (2014). Oral antiviral therapy for prevention of genital herpes outbreaks in immunocompetent and nonpregnant patients. Cochrane Database Syst Rev.

[3] Kimberlin DW, Rouse DJ (2004). Clinical practice. Genital herpes. N Engl J Med.

[4] Johnston C, Corey L (2016). Current concepts for genital Herpes Simplex Virus Infection: diagnostics and pathogenesis of genital tract shedding. Clin Microbiol Rev.

[5] Lebrun-Vignes B, Bouzamondo A, Dupuy A, Guillaume JC, Lechat P, Chosidow O (2007). A meta-analysis to assess the efficacy of oral antiviral treatment to prevent genital herpes outbreaks. J Am Acad Dermatol.

[6] Kukhanova MK, Korovina AN, Kochetkov SN (2014). Human herpes simplex virus: life cycle and development of inhibitors. Biochemistry (Mosc).

[7] Mattison HR, Reichman RC, Benedetti J (1988). Double-blind, placebo-controlled trial comparing long-term suppressive with short-term oral acyclovir therapy for management of recurrent genital herpes. Am J Med.

[8] Corey L, Wald A, Patel R (2004). Once-daily valacyclovir to reduce the risk of transmission of genital herpes. N Engl J Med.

[9] Yavuz BB, Cankurtaran M, Halil M, Dagli N, Kirkpantur A (2005). Renal dysfunction after oral acyclovir treatment in a geriatric woman: a case report. Scand J Infect Dis.

[10] Becker BN, Fall P, Hall C, Milam D, Leonard J, Glick A, Schulman G (1993). Rapidly progressive acute renal failure due to acyclovir: a case report and review of the literature. Am J Kidney Dis.

[11] Berry L, Venkatesan P (2014). Aciclovir-induced neurotoxicity: utility of CSF and serum CMMG levels in diagnosis. J Clin Virol.

[12] Lam NN, Weir MA, Yao Z (2013). Risk of acute kidney injury from oral acyclovir: a population-based study. Am J Kidney Dis.

[13] Reichman RC, Badger GJ, Mertz GJ (1984). Treatment of recurrent genital herpes simplex infections with oral acyclovir. JAMA.

[14] Tyring S, Wald A, Zadeikis N, Dhadda S, Takenouchi K, Rorig R (2012). ASP2151 for the treatment of genital herpes: a randomized, double-blind, placebo- and valacyclovir-controlled, dose-finding study. J Infect Dis.

[15] Aoki FY, Tyring S, Diaz-Mitoma F, Gross G, Gao J, Hamed K (2006). Single-day, patient-initiated famciclovir therapy for recurrent genital herpes: a randomized, double-blind, placebo-controlled trial. Clin Infect Dis.

[16] Cernik C, Gallina K, Brodell RT (2008). The treatment of herpes simplex infections: an evidence-based review. Arch Intern Med.

[17] Reitano M, Tyring S, Lang W (1998). Valaciclovir for the suppression of recurrent genital herpes simplex virus infection: a large-scale dose range-finding study. International valaciclovir HSV study group. J Infect Dis.

[18] Mertz GJ, Loveless MO, Levin MJ, Kraus SJ, Fowler SL, Goade D, Tyring SK (1997). Oral famciclovir for suppression of recurrent genital herpes simplex virus infection in women. A multicenter, double-blind, placebo-controlled trial. Collaborative famciclovir genital herpes research group. Arch Intern Med.

[19] Polansky H, Javaherian A (2016). 3-Ecosystems: microRNAs, receptors, and latent viruses: some insights biology can gain from economic theory. Front Microbiol.

[20] Polansky H, Javaherian A (2015). Commentary: the unliganded glucocorticoid receptor positively regulates the tumor suppressor gene BRCA1 through GABP beta. Front Cell Infect Microbiol.

[21] Polansky H (2003). Microcompetition with foreign DNA and the origin of chronic disease.

[22] Polansky H, Itzkovitz E (2013). Gene-Eden-VIR is antiviral: results of a post marketing clinical study. Pharmacol Pharm.

[23] Polansky H, Itzkovitz E (2014). Gene-Eden-VIR decreased physical and mental fatigue in a post marketing clinical study that followed FDA guidelines; results support microcompetition theory. Pharmacol Pharm.

[24] Polansky H, Javaherian A, Itzkovitz E (2016). Clinical study in genital herpes: natural Gene-Eden-VIR/Novirin versus acyclovir, valacyclovir, and famciclovir. Drug Des Devel Ther.

[25] US Department of Health and Human Services, Food and Drug Administration, Center for Drug Evaluation and Research (CDER), “Guidance for Industry, E 10 Choice of Control Group and Related Issues in Clinical Trials,” 2001

[26] Calvert M, Kyte D, Duffy H (2014). Patient-reported outcome (PRO) assessment in clinical trials: a systematic review of guidance for trial protocol writers. PLoS ONE.

[27] Vodicka E, Kim K, Devine EB, Gnanasakthy A, Scoggins JF, Patrick DL (2015). Inclusion of patient-reported outcome measures in registered clinical trials: evidence from ClinicalTrials.gov (2007–2013). Contemp Clin Trials.

[28] Willke RJ, Burke LB, Erickson P (2004). Measuring treatment impact: a review of patient-reported outcomes and other efficacy endpoints in approved product labels. Control Clin Trials.

[29] Albrecht MA. Clinical features (2015). In: Epidemiology, clinical manifestations, and diagnosis of genital herpes simplex virus infection, UpToDate. http://www.uptodate.com/home

[30] Romanowski B, Marina RB, Roberts JN (2003). Valtrex HS230017 study group. patients’ preference of valacyclovir once-daily suppressive therapy versus twice-daily episodic therapy for recurrent genital herpes: a randomized study. Sex Transm Dis.

