# Expression of Concern to: Clinical study of Gene-Eden-VIR/Novirin in genital herpes: suppressive treatment safely decreases the duration of outbreaks in both severe and mild cases

**DOI:** 10.1186/s40169-019-0222-0

**Published:** 2019-02-06

**Authors:** Hanan Polansky, Edan Itzkovitz, Adrian Javaherian

**Affiliations:** The Center for the Biology of Chronic Disease (CBCD), 616 Corporate Way, Suite 2-3665, Valley Cottage, NY 10989 USA

## Expression of Concern: Clin Trans Med (2016) 5:40 10.1186/s40169-016-0121-6

The Editor of this journal has published this Expression of Concern to alert readers that, following publication of the article [1], it came to light that there were undeclared competing interests that have a direct impact on the article and the strength of its findings. In addition, post publication peer review has indicated that the conclusions may not be supported by the results due to methodological flaws in the study. Pending further investigation of these concerns, further editorial action may be taken. The authors have not responded to any correspondence about this Expression of Concern.
